# Ag/Mo Doping for Enhanced Photocatalytic Activity of Titanium (IV) Dioxide during Fuel Desulphurization

**DOI:** 10.3390/molecules29194603

**Published:** 2024-09-27

**Authors:** Zahraa A. Hamza, Jamal J. Dawood, Murtadha Abbas Jabbar

**Affiliations:** 1 Department of Materials Engineering, University of Technology-Iraq, Bagdad 10066, Iraq; 130015@uotechnology.edu.iq; 2 Department of Mechanical Engineering, College of Engineering, University of Basrah, Basrah 61004, Iraq; murtadha.jabbar@uobasrah.edu.iq

**Keywords:** photodesulphurization, TiO_2_, double doping, silver and molybdenum, light cut

## Abstract

Regarding photocatalytic oxidative desulphurization (PODS), titanium oxide (TiO_2_) is a promising contender as a catalyst due to its photocatalytic prowess and long-term performance in desulphurization applications. This work demonstrates the effectiveness of double-doping TiO_2_ in silver (Ag) and molybdenum (Mo) for use as a novel catalyst in the desulphurization of light-cut hydrocarbons. FESEM, EDS, and AFM were used to characterize the morphology, doping concentration, surface features, grain size, and grain surface area of the Ag/Mo powder. On the other hand, XRD, FTIR spectroscopy, UV-Vis, and PL were used for structure and functional group detection and light absorption analysis based on TiO_2_’s illumination properties. The microscopic images revealed nanoparticles with irregular shapes, and a 3D-AFM image was used to determine the catalyst’s physiognomies: 0.612 nm roughness and a surface area of 811.79 m^2^/g. The average sizes of the grains and particles were calculated to be 32.15 and 344.4 nm, respectively. The XRD analysis revealed an anatase structure for the doped TiO_2_, and the FTIR analysis exposed localized functional groups, while the absorption spectra of the catalyst, obtained via UV-Vis, revealed a broad spectrum, including visible and near-infrared regions up to 1053.34 nm. The PL analysis showed luminescence with a lower emission intensity, indicating that the charge carriers were not thoroughly combined. This study’s findings indicate a desulphurization efficiency of 97%. Additionally, the promise of a nano-homogeneous particle distribution bodes well for catalytic reactions. The catalyst retains its efficiency when it is dried and reused, demonstrating its sustainable use while maintaining the desulphurization efficacy. This study highlights the potential of the double doping approach in enhancing the catalytic properties of TiO_2_, opening up new possibilities for improving the performance of photo-oxidative processes.

## 1. Introduction

Photocatalytic oxidative desulphurization (PODS) has recently attracted significant attention as an effective method for removing sulphur from various sources. Unlike traditional techniques such as hydrodesulphurization, PODS operates at lower temperatures and pressures and does not require hydrogen gas [1,2]. It can effectively reduce the sulphur content in fuel that contains thiophene or dibenzothiophene by selectively oxidizing them with photocatalysts, and it contributes to a cleaner air quality, as it reduces SOx emissions [3]. PODS requires the use of catalysts, with metal oxides, doped semiconductors, ionic liquids, and metal–organic frameworks all having been effective in eliminating sulphur compounds [4]. However, most semiconductor catalysts absorb only a tiny fraction of the solar spectrum, necessitating further development of catalysts that can utilize visible light. Desulphurization reactions have been conducted with titania-based catalysts, exhibiting potential in eliminating sulphur-containing formulations from fuel, in addition to demonstrating stability and low toxicity. Several studies have investigated the use of titania-based catalysts for photocatalytic oxidative desulphurization (PODS) processes [5]; for example, a C/TiO_2_@MCM-41 nanoparticle catalyst was found to efficiently remove sulphur-containing compounds within a short time [6]. The photocatalytic activity of Cu–Fe/TiO_2_ was proven to be superior to that of a photocatalyst made solely of TiO_2_ [7]. Furthermore, these catalysts have shown encouraging results in effectively removing sulphur-containing compounds from model fuels, achieving a minimal sulphur content, including Pt-decorated carbon-doped TiO_2_, a microporous titania–silica nanocomposite, and Co-TiO_2_/g-C3N4 ternary heterojunctions [8,9,10]. These studies have highlighted the importance of titanium dioxide as a support material for desulphurization. Because of its comparatively wide bandgap energy (about 3.2 eV), TiO_2_ can only absorb solar radiation in the ultraviolet spectrum. Its use in solar-driven photocatalysis is limited by this energy gap [11].

Researchers have discovered that adding metals or non-metals to a TiO_2_ structure or doping the TiO_2_ structure with metals or non-metals can improve its catalytic properties. This enhancement facilitates the oxidation of sulphur in hydrocarbons, thereby transforming it into forms that are less harmful to the environment [12]. Researchers have conducted extensive research on the mechanism of modification and the correlation between the crystalline structure and the catalytic activity of TiO_2_ doped with transition and noble metal ions. Silver and molybdenum dopants were found to improve visible light absorption and inhibit electron–hole recombination, which is beneficial for effectively removing sulphur. As a result, in this study, the efficacy of using double-doped Ag/Mo-TiO_2_ to desulphurize petroleum is investigated. Additionally, altering its structure using metals or non-metals via incorporation or doping enhances its catalytic properties, facilitating the oxidation of sulphur in hydrocarbons and transforming the petroleum into less environmentally harmful forms [13,14]. Researchers have extensively studied and reviewed the mechanism of modification, as well as the relationship between the crystalline structure and catalytic activity, for TiO_2_ doped with transition and noble metals. Silver and molybdenum dopants allow visible light to pass through and slow down the electron–hole grouping, which aids in the elimination of sulphur reactions. Therefore, in this study, we explore the performance of double-doped Ag/Mo-TiO_2_ when desulphurizing petroleum.

## 2. Results and Discussion

### 2.1. Characterization

#### 2.1.1. Field Emission Scanning Electron Microscopy (FESEM) and Particle Size

**Figure 1:**
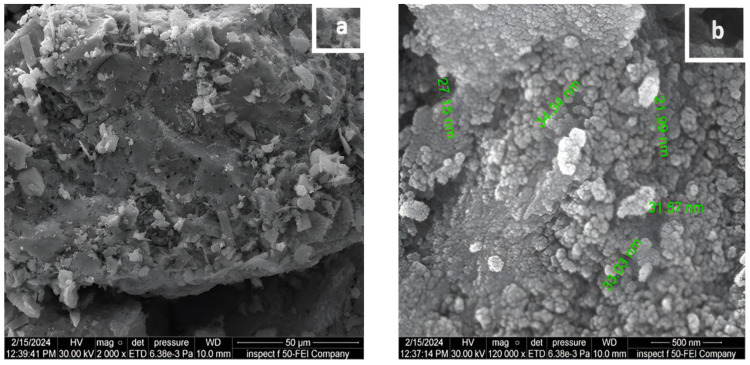
Micrograph of the Ag/Mo-doped TiO_2_ powder. (**a**) Catalysed powder particles; (**b**) discrete particle size.

The morphology of the Ag/Mo powder was investigated via field emission scanning electron microscopy (FESEM), which provided insights into the surface structure and particle size of the photocatalysts. The prepared photocatalyst appeared to be agglomerated and had a uniform surface with semi-spherical particles, according to the FESEM images presented in Figure 1a,b. The doped particles were evenly dispersed and exhibited a modest size, with an estimated particle size range of 22 to 34 nm for the Ag/Mo-TiO_2_, as shown in Figure 1b.

**Figure 2:**
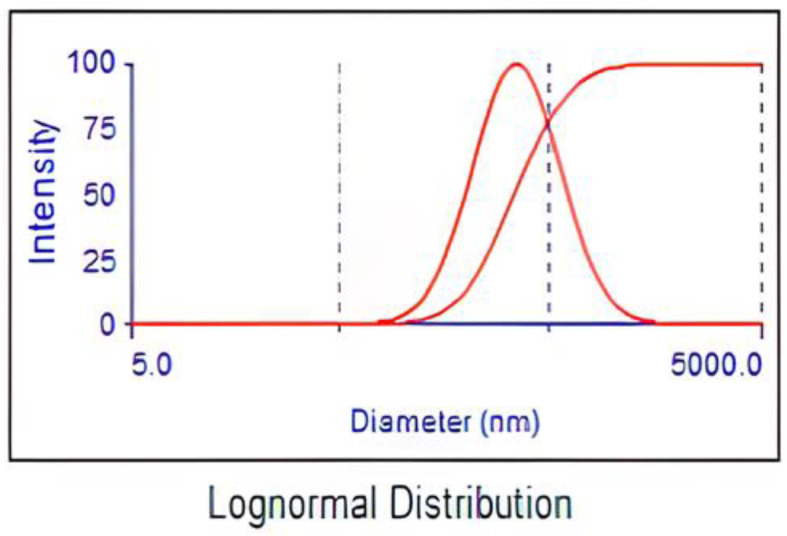
Particle size distribution of Ag/Mo-doped TiO_2_.

In Figure 1a, the catalyst exhibits multiple pores on its surface, and the Ag and Mo are distributed in a non-uniform way. Figure 2 displays a histogram of the distributed nanoparticles. The average particle size was determined to be 344.4 nm, with a dispersion factor of 0.254. The sol–gel method precisely controlled the particle size and distribution, which led to a moderate dispersion factor that worked well with the photocatalyst’s features [15,16]. This uniform dispersion ensures that the dopants are effectively integrated into the TiO_2_ matrix.

#### 2.1.2. Atomic Force Microscopy

**Figure 3:**
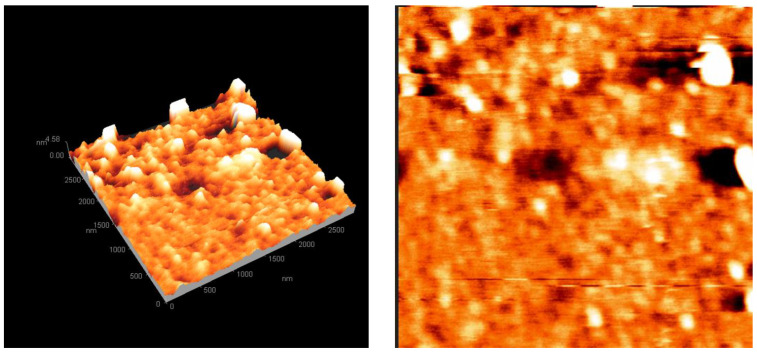
Three- and two-dimensional AFM images of the deposited Ag/Mo-doped TiO_2_ powder.

Figure 3 displays mode-of-rhythm AFM micrographs of the doped TiO_2_ thin films deposited on a glass substrate via spin coating. The average roughness and grain size of the Ag/Mo-TiO_2_ were 0.612 and 32.15 nm, respectively, with a root mean square roughness of 0.889 nm. The incorporation of Ag and Mo into the TiO_2_ lattice was responsible for the mild surface roughness [17,18]. The sample’s skewness value was −0.079, indicating more troughs than peaks. This negative skewness meant that the surface had more depressions, which made it more wettable and less hydrophobic, allowing the light distillate to interact with it better [19].

Active catalytic sites are often found in surface irregularities. The negative skewness thus improved the catalytic reactivity [20]. The doped TiO_2_ achieved a large particle surface area of 811.79 nm² and an average height of 0.63 nm, which increased the likelihood of successful collisions between the reactant molecules. The reduced average height facilitated effective access to the active sites, enhancing the catalyst’s efficiency in desulphurization and other catalytic approaches [21].

#### 2.1.3. Energy-Dispersive X-ray Spectroscopy (EDS)

**Figure 4:**
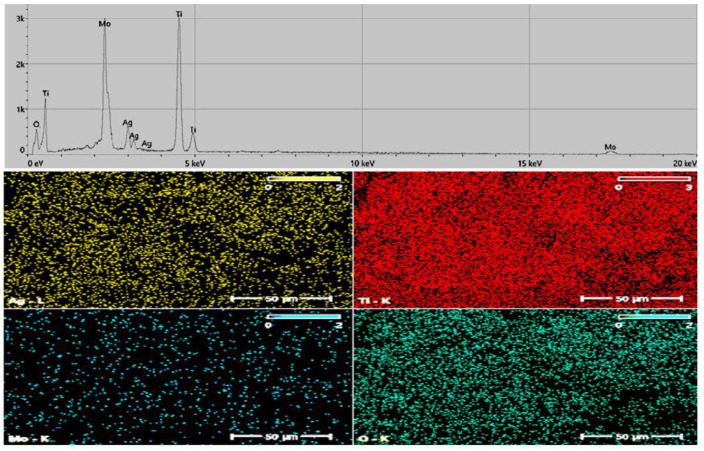
EDS spectrum with mapping of the Ag/Mo-doped TiO_2_ catalyst.

Figure 4 presents the energy-dispersive X-ray spectroscopy (EDS) spectra of the doped titanium dioxide (TiO_2_) samples. The results indicate that the proportions of doped TiO_2_ varied, with the mapping showing the highest concentration of Ti in the catalyst. The abundance of silver (Ag) was significantly lower than that of titanium (Ti), while molybdenum (Mo) had the lowest concentration. The predicted percentage was achieved when Ag and Mo were successfully doped into the TiO_2_ structure.

#### 2.1.4. X-ray Diffraction Analysis

**Figure 5:**
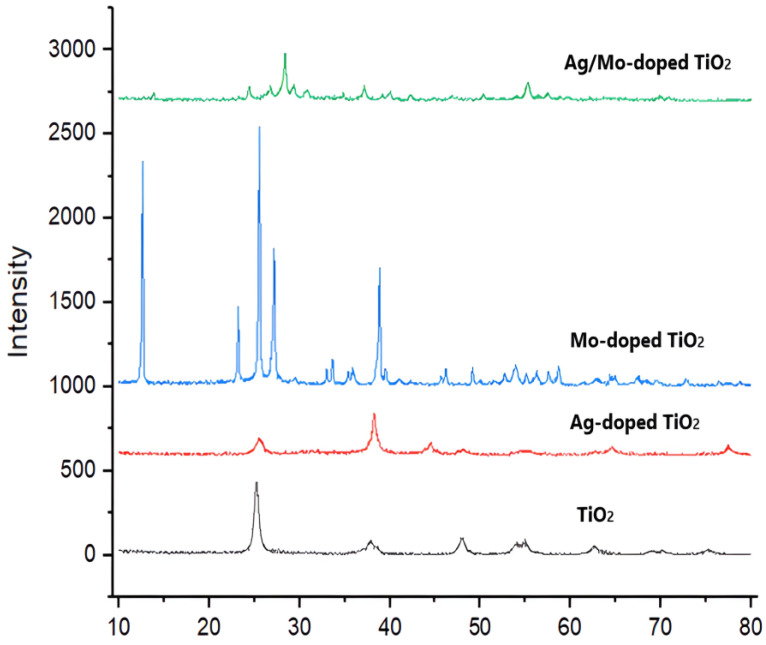
X-ray diffraction of TiO_2_, Ag-doped TiO_2_, Mo-doped TiO_2_, and Ag/Mo-doped TiO_2_.

The XRD patterns of the four different samples are shown in Figure 5. These samples were (a) pure TiO_2_, (b) Ag-doped TiO_2_, (c) Mo-doped TiO_2_, and (d) Ag/Mo-doped TiO_2_. With the introduction of the dopants, the film’s characteristic peaks changed. The Joint Committee on Powder Diffraction Standards (JCPDS card no. 21-1272) determined that the pure TiO_2_ samples had unique summits that matched the anatase phase of TiO_2_ and had tetragonal structures.

The original 2θ angles of the pure TiO_2_ peaks (25.26° and 37.84°) moved to higher angles (25.57° and 38.2°) in the Ag-doped samples, indicating that the lattice spacing decreased [22,23]. The nano-sized Ag doping induced compressive strain within the crystal lattice, leading to decreased lattice parameters [24,25]. Conversely, the third peak (2θ angle: 47.97°) exhibited a lower angle (44°), suggesting lattice expansion.

In Mo-doped TiO_2_, the 2θ angles shifted to higher values (12.686°, 25.57°, and 27.1988°), indicating an increase in lattice spacing. Mo doping introduced tensile strain, causing lattice expansion and a potential reduction in the lattice parameters. The XRD peaks of Ag/Mo-doped TiO_2_ were moved to higher (28.3897° and 37.17°) and lower (55.32°) angles than those of the pure TiO_2_. The shift to higher angles suggests reduced lattice spacing, whereas the shift to a lower angle indicates lattice expansion. The molybdenum atoms substituting some of the Ti atoms introduced strain, leading to lattice expansion, whereas the Ag atoms were associated with the TiO_2_ atom’s surface. The presence of two peaks indicating compressive strain suggests that Ag ions function similarly to Mo ions. This double-doped TiO_2_ system might have a better photocatalytic performance since the addition of Ag changes the lattice strain.

#### 2.1.5. Fourier Transform Infrared (FTIR) Spectroscopy

**Figure 6:**
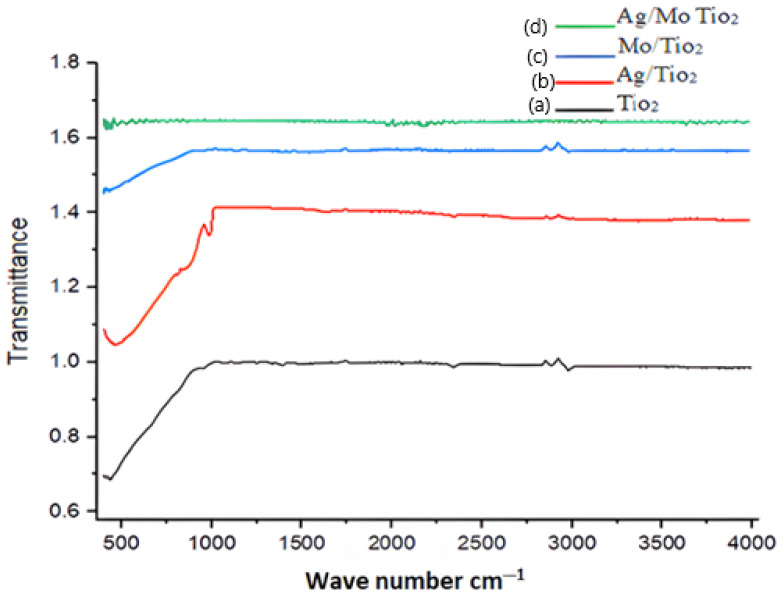
FTIR spectra of (**a**) TiO_2_, (**b**) Ag-doped TiO_2_, (**c**) Mo-doped TiO_2_, and (**d**) Ag/Mo-doped TiO_2_.

In Figure 6a, the Fourier transform infrared (FTIR) spectroscopy transmittance data obtained for the prepared TiO_2_ correspond closely with the reference spectrum for TiO_2_ (anatase) sourced from the NIST Chemistry WebBook [26]. This reference spectrum represents pure anatase TiO_2_, denoted by the O=Ti=O structure. Impurities, such as hexamethylene amine, could potentially cause discrepancies in the data.

Key features observed in the FTIR data include peaks and fluctuations across various wavelengths. Anatase TiO_2_ typically demonstrates distinctive vibrational modes or strong absorption bands around 400 cm^−1^, primarily ascribed to Ti-O stretching modes. Additional bands may arise from lattice vibrations and surface modes, contributing to the overall spectrum [27].

The transmittance values observed for Ag-doped TiO_2_ (Figure 6b) ranged from approximately 0.88 to 0.93. Notable wavenumbers such as 3989, 3837, and 3801 cm^−1^ correspond to Ti-O stretching vibrations, with 3751 cm^−1^ potentially associated with lattice vibrations or surface modes due to Ag ion integration. The region around 3650 cm^−1^ typically involves bending modes or lattice vibrations, while the 2981 cm^−1^ region may relate to catalyst functional groups created by lattice doping [28]. Additionally, the presence of peaks at 1772 cm^−1^ suggests C=O stretching vibrations, indicating the presence of organic impurities. Finally, the peak at 588 cm^−1^ is often associated with lattice vibrations or Ti-O modes.

In Figure 6c, the high wavenumber peak at 3386 cm^−1^ may correspond to Ti-O stretching vibrations within the Mo-doped TiO_2_ lattice. Mo doping introduces additional peaks at 2878, 2816, and 2345 cm^−1^, while Ti-O stretching vibrations might subsidize the peak at 463 cm^−1^ [29]. The addition of Ag and Mo dopants may change these peaks. Ag may have added more vibrational modes, especially in the high wavenumber region, while Mo altered the lattice vibrations and overall spectral characteristics. The transmittance values for Mo-doped TiO_2_ ranged from approximately 0.62 to 0.67.

The data shown in Figure 6d indicate high transmittance values across the full spectrum, ranging from approximately 0.99 to 1.0. It is likely that the peak at 3648.62 cm^−1^ is caused by OH (Ti-OH) stretching vibrations, which are functional groups that are key for desulphurization [30,31]. The peaks at 2061.61 and 2037.97 cm^−1^, observed at high transmittance, indicate vibration and stretching in the lattice due to double doping. Low transmittance peaks at wavenumbers of 474.27, 466.79, 444.63, 430.29, and 414.62 cm^−1^ are usually linked to metal oxide lattice vibrations.

#### 2.1.6. Optical Properties

**Figure 7:**
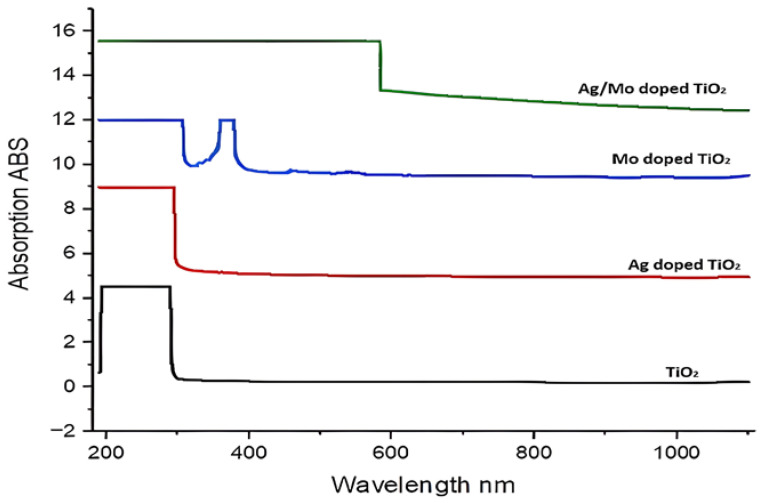
UV-Vis spectra of TiO_2_, Ag-doped TiO_2_, Mo-doped TiO_2_, and Ag/Mo-doped TiO_2_.

The ultraviolet–visible (UV-Vis) absorption spectra of the four different TiO_2_ photocatalysts are shown in Figure 7. The absorption edge for TiO_2_ falls within the range of 300–350 nm, and the peaks indicate the excitation of electrons from the valence band to the conduction band. This behaviour is characteristic of anatase TiO_2_ and indicates its primary UV light absorption. Doping Ag into TiO_2_ causes a slight shift compared to pure TiO_2_. The absorption values are higher than those of TiO_2_, especially in the visible range, suggesting that Ag doping improved TiO_2’_s light absorption capacity. Mo-TiO_2_ exhibits absorption that extends into longer wavelengths, up to approximately 1022.43 nm. Furthermore, Ag/Mo-TiO_2_ exhibits significantly higher absorption across all the measured wavelengths, from the visible to infrared regions, up to about 1053 nm. Subsequently, energy bandgaps were calculated by Equation (1) based on absorbed wavelengths and related standards parameters (Table 1) [32].

The bandgaps were calculated using Equation (1) as follows:

(1) E=hc/λ

where: h, c, λ are Plancks ’constant, light speed and wavelength correspondingly.

Anatase’s bandgap is around 3.54 eV, which means that it only absorbs UV light from the solar spectrum due to its large band energy [33,34]. Ag adds new electronic states, thus lowering the bandgap. It can also cause localized surface plasmon resonance (LSPR), which increases the electromagnetic field in the area [35,36]. This improves the photocatalytic activity by enhancing light absorption, particularly under visible light. Mo doping introduces new energy levels within the bandgap, effectively narrowing it. Mo can generate mid-gap states and new energy levels that encourage the absorption of visible light [37,38,39]. When Ag and Mo are co-doped, they create new electronic states within the bandgap or significantly modify its width; the highest absorption values among all the samples indicate the most effective light absorption. The combination of Ag and Mo doping creates synergistic effects, with Ag providing plasmonic enhancement and Mo introducing new energy levels. This combination results in the best charge carrier dynamics and light absorption. Potentially, a longer range of wavelengths could enhance the penetration, irradiating the entire fuel volume and enhancing the photoactivity for desulphurization [40]. This increased photoactivity makes the photocatalyst more reactive, as it absorbs longer-range wavelengths, ultimately enhancing its overall effectiveness in desulphurization.

#### 2.1.7. Photoluminescence (PL)

**Figure 8:**
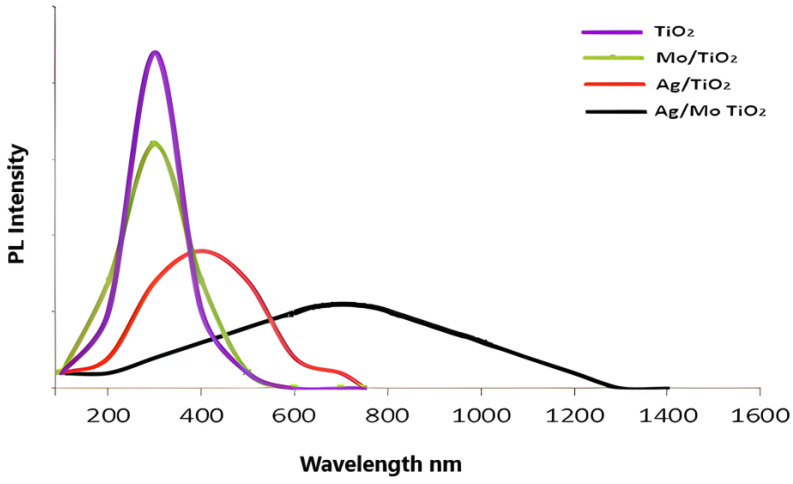
PL spectra of the catalysts.

In photocatalytic materials, photoluminescence (PL) spectroscopy is used to investigate the electronic structure and the recombination rate of electrons and holes. Figure 8 displays the photoluminescence (PL) emission spectra of the undoped TiO_2_, 5% Ag-TiO_2_, 2% Mo-TiO_2_, and 5% Ag/2% Mo-TiO_2_ photocatalysts.

The photocatalysts exhibited different emission spectra in various nm ranges. Nevertheless, the photoluminescence (PL) intensities of both 5% Ag-TiO_2_ and Ag/Mo-TiO_2_ were significantly reduced compared to those of pure TiO_2_ and 2% Mo-TiO_2_. The PL emission intensity is directly related to the recombination of electron–hole pairs in the TiO_2_ photocatalyst [41]. A lower photoluminescence (PL) intensity means that photo-excited electrons and holes are recombining more slowly, which is good for the photocatalytic process. The interaction between silver (Ag) and molybdenum (Mo) can result in a synergistic effect, causing alterations in energy levels and the dynamics of the charge carriers. This combined effect may make the photoluminescence (PL) less bright because there are more recombination centres (Ag) and localized states (Mo) [42]. For this reason, the Ag particles on the surface of TiO_2_ can effectively catch and store the photo-excited electrons. Additionally, Mo can stop e–h recombination by creating localized electronic states close to the band edges. These states facilitate radiative recombination, leading to increased charge carrier lifetimes.

### 2.2. Photodesulphurization

**Figure 9:**
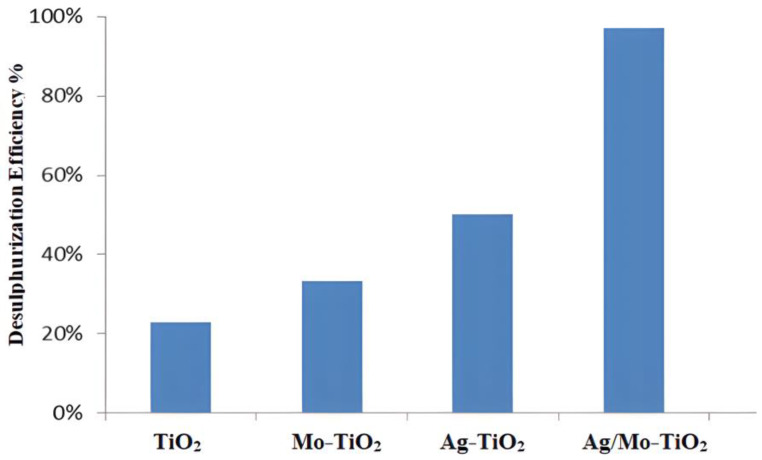
Desulphurization efficiency of TiO_2_ catalysts.

Pure TiO_2_ has a limited desulphurization efficiency of approximately 23%, as seen in Figure 9, due to its relatively large bandgap (~3.54 eV). This restricts its absorption of UV light, and it also has a high PL intensity, as shown in the PL results, which means that the electrons and holes are recombining quickly [43]. This makes fewer charge carriers available for the desulphurization reaction. Mo-doped TiO_2_ increases the efficiency of desulphurization to 34% by changing the bandgap (2.7 eV) and adding new energy levels within the bandgap, which improves the charge carrier dynamics. However, the addition of 5% silver (Ag) significantly increases the desulphurization efficiency to 51% by extending the light absorption into the infrared range, as seen in Figure 7, and improving the charge separation at low PL intensities, as indicated by Figure 8. This is due to plasmonic effects and reduced electron–hole recombination, although Mo-doped TiO_2_ may not provide the same level of plasmonic enhancement as Ag doping [44]. Similarly, when it is exposed to light, Ag/Mo-TiO_2_ becomes more active, especially when it absorbs and emits 778 nm at low intensities. The wide absorption range and decreased recombination rates lead to a higher concentration of active charge carriers. These carriers can effectively take part in the redox reactions needed for desulphurization, streamlining the breakdown of sulphur-containing compounds. When the catalyst absorbs photons, electrons in the valence band are excited to the conduction band, leaving behind holes in the valence band. This process creates electron–hole pairs, which are vital for photocatalytic reactions [45]. The presence of Ag and Mo aids in reducing the recombination rate of electron–hole pairs. Ag nanoparticles act as electron traps, while Mo introduces new energy levels that enable better charge separation. The separated electrons and holes move to the surface of the TiO_2_ particles, where they participate in redox reactions. The photogenerated holes (h^+^) on the surface of the TiO_2_ can oxidize sulphur-containing compounds (e.g., thiols and sulphides) to produce sulphur oxides (SO_x_) or other oxidized sulphur compounds. Light (e-) electrons can break down the oxygen molecules stuck to the catalyst surface to produce reactive oxygen species (ROS), which include superoxide radicals (O^−2^) and hydroxyl radicals (^•^OH), to then be expelled [46]. The sulphur content was quantified using Equation (1), revealing a desulphurization efficiency of about 97%.

**Figure 10:**
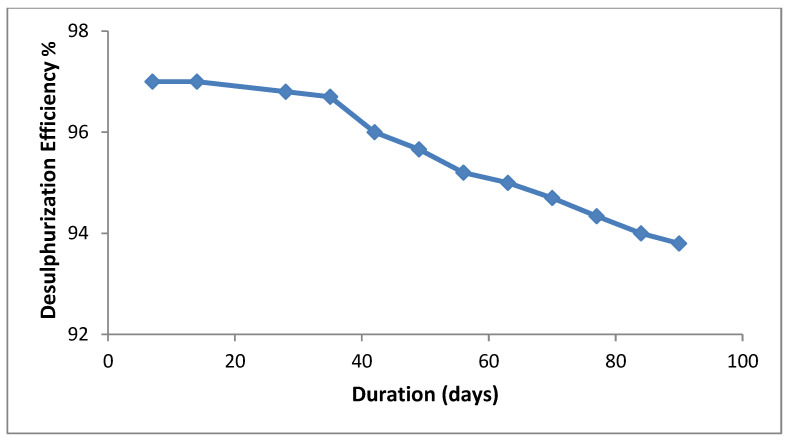
Desulphurization efficiency of the catalysts with exposure over time.

The large surface area of the particles obtained (811.79 nm^2^) improves adsorption and allows more sulphur-containing molecules to be attracted to the catalytic surface. This also increases the number of active sites where photogenerated electrons and holes can participate in redox reactions [47]. As a result, the efficiency of photodesulphurization is significantly improved, as the catalyst can efficiently adsorb and react with the sulphur. Nanoscale particles have quantum effects that become significant when the electron–hole mobility is limited and hinder the recombination within small particles to enhance their reactivity. They absorb visible light due to their quantum confinement effect, expanding the range of wavelengths for photodesulphurization [48]. As the temperature gradually increased (from 40 °C to 70 °C), the desulphurization efficiency declined due to an excess of electrons and the recombination of electron–hole pairs stemming from the narrow bandgap of the catalyst [49]. However, the catalyst was recycled for three months, and we intermittently examined the sulphur concentrations, noting that the efficiency decreased by 3.4%, as displayed in Figure 10. The catalyst retained its efficiency upon drying and reuse, demonstrating its sustainable use while maintaining the desulphurization efficacy.

## 3. Experiments

To investigate double doping in TiO_2_, four catalysts were prepared following the sol–gel method using titanium (IV) isopropoxide (TTIP) (97%) (Sigma Aldrich, CAS No. 546-68-9), silver nitrate (Daejung Chemicals, Busan, Republic of Korea, CAS No. 7761-88-8), ammonium molybdate (Thomas Baker, Mumbai, India, CAS No. 12054-85-2), sodium borohydride (99%) (Sigma Aldrich, St. Louis, MA, USA, CAS No. 16940-66-2), and hexamethylenetetramine (Thomas Baker Chemicals Pvt. Ltd., Mumbai, India, CAS No. 100-97-0). Distilled water, ethanol (99%), 97% nitric acid, and 37% hydrogen peroxide were used for oxidation.

### The Sol–Gel Method

A mixture of ethanol and TTIP at a 3:1 ratio was stirred with a magnetic stirrer, and a few drops of nitric acid were added to adjust the pH to 1. Distilled water was then gradually added until titanium oxide particles formed, resulting in a white emulsion; this was then stirred at 80 °C for 3 h and aged for 12 h.

After the gel formed, it was washed, dried at 100 °C in the furnace, and then calcined at 400 ℃ for four h. TiO_2_ was doped with Ag and Mo separately following the same procedure: 5 M silver nitrate and 2 M ammonium molybdate solutions were added, and then the resultant solution was stirred, aged for 24 h, reduced using NaBH4, and calcinated for 2 h at 500 °C. To dope silver and molybdenum into TiO_2_, we repeated the process and stirred the same ratio of TTIP to ethanol and the drops of nitric acid into the mixture for 30 min at 80 °C.

**Figure 11:**
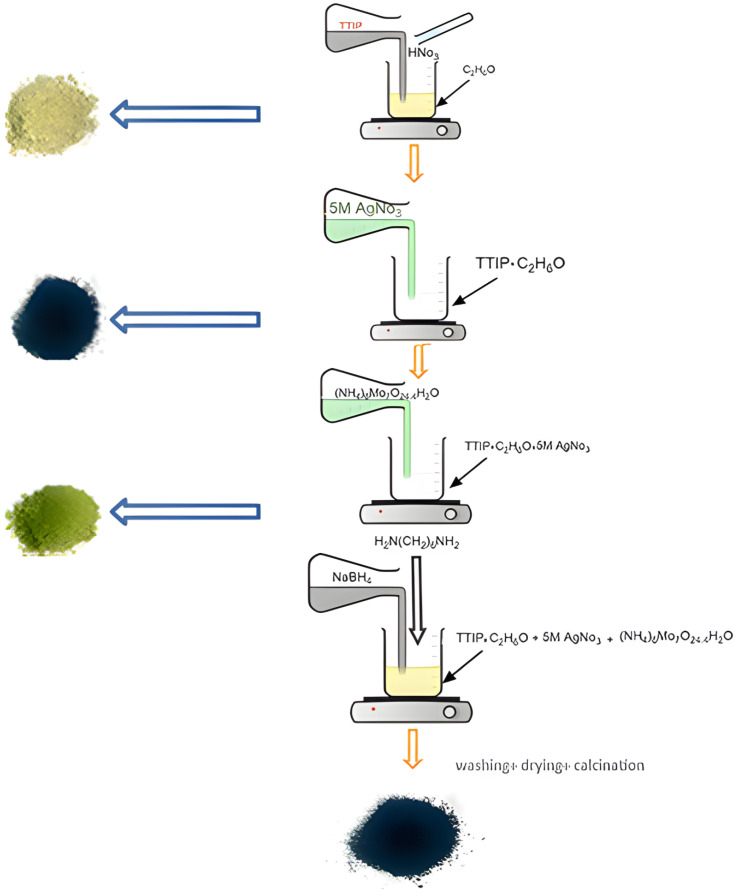
Sol–gel method used to prepare the Ag/Mo-doped TiO_2_.

The solution was stirred for 3 h after the addition of distilled water, after which a 5 M silver nitrate solution was introduced to form a homogenous slurry. Subsequently, a 2 M ammonium molybdate solution was added. Sodium borohydride was also used for reduction, which turned the mixture black. Hexamethylenetetramine was added to all the synthesized powders to improve the dispersion. The resultant mixture was stirred for 5 h and aged for 24 h. The gel then formed, was washed several times with distilled water, was dried at 100 °C for 2 h, was calcined at 550 °C for 2 h, and was allowed to cool slowly. Figure 11 illustrates these steps.

The catalysts underwent analysis using various techniques. X-ray diffraction (XRD) was carried out with an Empyrean XRD instrument (Huston, TX, USA) using Cu (1.54060 Å) with a voltage of 40.0 kV, a current of 30.0 mA, a scan range of 10–80°, and a step size of 0.0400°.

Fourier transform infrared (FTIR) spectra were acquired using a TENSOR-27/Bruker spectrometer (Billerica, MA, USA). The elemental composition was determined via energy-dispersive spectroscopy (EDS, Thermo Fisher Scientific, Waltham, MA, USA). A field emission scanning electron microscope (Inspect F 50 FEI, FEI Company, Eindhoven, The Netherlands) and an atomic force microscope (AFM, Suwon, Republic of Korea) were used to investigate the particle size, surface features, roughness, and surface area. To achieve an agglomerated size distribution, a Brookhaven (Holtsville, NY, USA) particle size analyser was employed. The substance was thoroughly dispersed in distilled water using an ultrasonicator and was analysed using 90Plus particle sizing software v3. UV–visible diffuse reflectance spectroscopy was performed using a spectrometer (SP-8001, Bruker, Billerica, MA, USA) within the wavelength range of 190–1100 nm. Photoluminescence (PL) spectra were obtained using a Shimadzu spectrofluorometer (Kyoto, Japan). A light cut (distillate), including heavy naphtha and kerosene, was supplied by the South Refineries Company (Basra, Iraq), with its specifications shown in Table 2.

The photocatalytic desulphurization reaction was evaluated by adding 0.7 g of the catalyst powder to 7 mL of the light cut in a dark box. Following that, 0.082 mL of hydrogen peroxide was dissolved in the mixture and stirred. In a dark container, the mixture was subsequently exposed to a 60-watt LED light bulb (simulating sunlight) under ambient conditions (25 °C) and atmospheric pressure (30, 45, and 60 min) and then filtered. The efficiency of the desulphurization was measured using a Petra sulphur analyser (Petra 4294) for petroleum and calculated using the following formula [50]:

(2) D=(x°−xf)/(x°)×100%

where *x^°^* and *x_f_* are the initial and final concentrations of sulphur, respectively.

After the specified practice durations, the photocatalytic activity dropped. To reactivate the catalyst, it was soaked in a 5% HNO_3_ solution for 2 h, then washed with distilled water, and dried. Later, the catalyst was dunked in ethanol to extract any impurities and left to dry.

## 4. Conclusions

A double-doped Ag/Mo-TiO_2_ catalyst was used, along with H_2_O_2_, to perform photocatalytic oxidative desulphurization (PODS), a method that effectively eliminates sulphur-containing compounds from light fuels. The results of the FTIR, XRD, AFM, and UV-Vis analyses showed that doping TiO_2_ with two types of elements changes its structure and optical properties in a way that improves its ability to speed up reactions. Ag/Mo doping introduced active sites, creating a highly efficient catalyst and narrowing the bandgap. Ag/Mo-doped TiO_2_ took in higher light wavelengths and experienced less electron–hole recombination, eliminating sulphur quickly and effectively 97% of the time. This suggests that such doped photocatalysts have the potential to effectively remove sulphur compounds from fuels while maintaining their ability over time. This catalyst’s ability to absorb a broad spectrum of light regions and its high desulphurization efficiency make it a promising candidate for industrial applications in refineries. This development can provide economic benefits by eliminating the need for elevated temperatures and pressures, thereby facilitating the process in tanks. The promise of a nano-homogeneous particle distribution with a high surface area also bodes well for future coating applications, offering a cleaner and more sustainable process.

## Figures and Tables

**Table 1:** Energy Band Gaps for TiO_2_ Catalysts.

**Energy band gap (e.v)**	**TiO_2_**	**Ag-Doped TiO_2_**	**Mo-Doped TiO_2_**	**Ag/Mo-Doped TiO_2_**
	3.54	1.23	2.7	1.18

**Table 2:** Specification of Light Distillate.

Light Distillate	Value
Specific gravity at 60 °C	0.733
Flash point	64
Viscosity	0.97
Sulphur content	892.5 ppm

## Data Availability

Data sharing is not applicable to this article, as no datasets were generated or analysed during this study.
